# Early Detection of Colon Cancer Following Incidental Finding of *Clostridium septicum* Bacteremia

**DOI:** 10.1177/2324709619832050

**Published:** 2019-03-12

**Authors:** Jasdeep Singh Sidhu, Amrendra Mandal, Jeevanjot Virk, Vijay Gayam

**Affiliations:** 1 Interfaith Medical Center, Brooklyn, NY, USA

**Keywords:** *Clostridium septicum*, colon carcinoma, bacteremia

## Abstract

*Clostridium septicum* is a Gram-positive, anaerobic, spore-forming bacillus found in the intestine. It is linked to colon cancer and immunosuppression. Infection with *C septicum* may vary in manifestation and is associated with more than 60% mortality rate. In this article, we present a case of incidental isolation of *C septicum* in a patient who presented with fever and later on colonoscopy was found to have colon carcinoma. Bacteremia may be the unexpected initial presentation of undiagnosed colon carcinoma.

## Introduction

Few bacterial infections have been associated with malignancy; one of them is *Clostridium septicum*, which is known to be associated with gastrointestinal (GI) and solid organ malignancy, immunosuppression, neutropenia, and diabetes mellitus.^1^ It has a variable clinical presentation, and if not treated early, it is associated with a high mortality with the majority of deaths occurring within the first 24 hours.^2^ We report a case of *C septicum* bacteremia associated with colon carcinoma. We understand that it is important for clinicians to be aware of clinicians of this rare but known association between colon cancer and *C septicum*.

## Case Presentation

A 71-year-old female with comorbidities such as hypertension and end-stage renal disease (on hemodialysis) presented to our emergency department with complain of back pain of 1 day duration. Only significant finding noted on physical examination was decreased range of motion in lumbar spine associated with moderate to severe pain. Review of systems was otherwise reported negative. The patient had low-grade fever 100.4°F, tachycardia 115 beats per minute, respiratory rate 22 breaths per minute, and blood pressure 160/78 mm Hg on admission. Complete blood count on admission was significant for macrocytic anemia with hemoglobin 10.1 mg/dL and mean corpuscular volume 101.3. Significant left shift was noted on differential white blood cell (WBC) count with 96.2% neutrophils (total WBC count on admission was 9500/dL). Comprehensive metabolic panel showed deranged renal function blood urea nitrogen 40 mg/dL (8-20 mg/dL) and creatinine 9.79 mg/dL (0.4-1.3 mg/dL). The patient met systemic inflammatory response syndrome criteria; therefore, 2 sets of blood cultures were sent for further sepsis workup and patient was started on broad-spectrum antibiotics (vancomycin and meropenem) empirically. Computed tomography chest/abdomen/pelvis was done, which was negative for any pulmonary or intra-abdominal focus of infection. However, computed tomography abdomen/pelvis showed presence of spinal canal stenosis in the lumbar area. Blood cultures sent on day 1 and day 3 (total 3 sets) grew *C septicum*. Repeat blood culture sent on day 5 was reported negative. Based on sensitivity report, antibiotics were switched to piperacillin/tazobactam.

Due to patient’s new-onset symptoms of worsening back pain, magnetic resonance imaging lumbar spine was done, which ruled out acute process/any mass lesion in the lumbar spine. Presence of multilevel degenerative disease and spinal canal stenosis was confirmed and any other pathology was ruled out.

**Figure 1.**
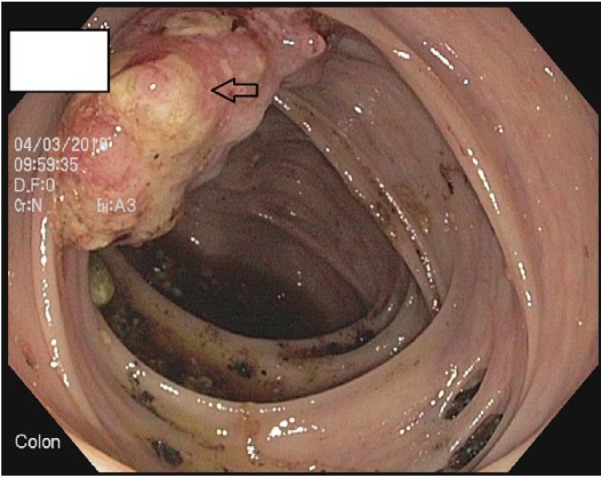
Colonoscopy showing nonobstructive mass in ascending colon suspicious for malignancy.

**Figure 2.**
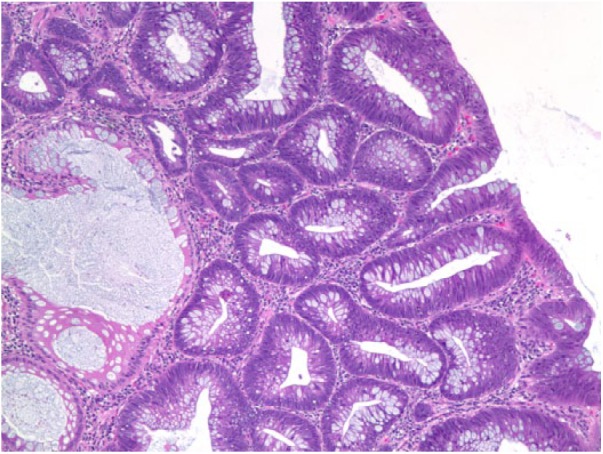
Histopathological section from the colonic mass showing adenocarcinoma.

The patient improved symptomatically and WBC count also improved over the next few days. However, due to known but rare association of *C septicum* with GI malignancy, upper GI endoscopy and colonoscopy (Figure 1) was performed on day 6, which showed nonobstructive mass in ascending colon suspicious for malignancy. Biopsy was taken and sent for histopathology examination, which was reported positive for well-differentiated adenocarcinoma (Figure 2).

As there was no metastasis, the patient underwent hemicolectomy for the aforementioned lesion in the ascending colon. She was followed-up in 3 months and underwent surveillance colonoscopy, which showed no local recurrence of the malignancy.

## Discussion

*Clostridium septicum* is a rare infection and is known to be associated with colorectal malignancy.^3^ Other similar association with colon cancer is *Streptococcus bovis*; however, as compared with the latter, *C septicum* is a lethal infection if not treated early. In one case review, among infection with *Clostridium* species, malignancy was found to be associated in 50% of patients with *C septicum*, whereas malignancy was seen in only 11% of patients with other clostridial infections and the remaining patients with spontaneous clostridial species infection all had evidence of immunosuppression.^4^

The clinical spectrum of *C septicum* varies and can present as cellulitis, fasciitis, myonecrosis, abscess, aortitis, or septic shock.^5^ There should be a high suspicion of colonic malignancy in the patients with *C septicum* bacteremia with or without underlying signs of sepsis. *C septicum* can present with nonspecific features such as fever, and the infection can be easily missed in asymptomatic patients with colon carcinoma.^6^ In the present case, we sent a blood culture as a part of sepsis workup without other clinical manifestations of colon cancer. Incidentally, *C septicum* infection was found leading to evaluation for the possible cause. The early initiation of treatment with antibiotics is also very important as sepsis-related mortality varies between 45% and 70%.^1,5^ This association between *C septicum* and colon cancer could be due to over proliferation of tumor tissue leading to relatively less blood supply with the development of tissue necrosis, which creates a favorable environment for anaerobic bacterial proliferation.

*Clostridium septicum* is more aerotolerant than *C perfringens* and therefore has predilection for normal tissue.^7^ There may be multiple virulence factors involved including production of several toxins and potential to aggressively invade the tissue.^8^ Tumor-induced mucosal ulceration results in disruption of the intact barrier, owing to translocation of the bacteria hematogenously from the bowel leading to sepsis. Once the malignancy outgrows its blood supply, the anaerobic environment created is ideal for bacterial growth.^5^ The infection spreads and tends to affect areas supplied by a single artery.^9^ Impaired host immunity as in our case from end-stage renal disease is also believed to facilitate translocation.

Cecal cancers are the most common locations linked with this infection likely due to characteristic pH, osmotic changes, and electrolyte changes conducive for multiplication of *C septicum*; however, tumor may be located at the other area such as ascending colon in our case.^7,10^ Mao et al observed that 4 patients who grew of *C septicum* in their blood cultures were also found to have colon cancer.^7^

In the present case, there was early hematogenous dissemination of *C septicum* from the colon carcinoma that led to early workup of malignancy and eventually colon carcinoma was diagnosed on colonoscopy. The patient was treated with antibiotics in a timely fashion and she underwent curative resection of colon carcinoma. Therefore, evidence of *C septicum* infection can be useful tool to detect any occult malignancy.

## Conclusions

The vigorous search for colorectal carcinoma should be done in any patient growing *C septicum* on blood culture with or without any symptoms.
